# Exploring the potential of *Hermetia illucens* larvae extracts: A promising approach for dermocosmetic formulations

**DOI:** 10.1016/j.heliyon.2024.e37395

**Published:** 2024-09-03

**Authors:** Márcia Santos Filipe, Rossana V.C. Cardoso, Manuel Ayuso, Daniel Murta, Ana María Díaz-Lanza, Catarina Rosado, Tânia C.S.P Pires, Ricardo C. Calhelha, Patricia Rijo

**Affiliations:** a CBIOS – Universidade Lusófona's Research Center for Biosciences & Health Technologies, Campo Grande 376, 1749-024, Lisbon, Portugal; b Universidad de Alcalá de Henares. Facultad de Farmacia, Departamento de Ciencias Biomédicas (Área de Farmacología, Nuevos agentes antitumorales, Acción tóxica sobre células leucémicasCtra. Madrid-Barcelona km. 33,600, 28805, Alcalá de Henares, Madrid, Spain; c Centro de Investigação de Montanha (CIMO), Instituto Politécnico de Bragança, Campus de Santa Apolónia, 5300-253, Bragança, Portugal; d Laboratório Associado para a Sustentabilidade e Tecnologia em Regiões de Montanha (SusTEC), Instituto Politécnico de Bragança, Campus de Santa Apolónia, 5300-253, Bragança, Portugal; e Ingredient Odyssey SA – EntoGreen, Rua Cidade de Santarém 140, 2005-079, Santarém, Portugal; f CiiEM - Centro de Investigação Interdisciplinar Egas Moniz, Campus Universitário, Caparica, Portugal; g Instituto de Investigação do Medicamento (iMed.ULisboa), Faculdade de Farmácia, Universidade de Lisboa, 1649-003, Lisbon, Portugal

**Keywords:** *Hermetia illucens*, Black soldier fly, Green economy, Extraction, Biological activity, Skin products

## Abstract

Globally, the yearly disposal of 1.3 billion tonnes of food raises environmental and public health concerns. Black soldier fly (BSF) larvae present a sustainable solution, converting organic waste into nutrient-rich biomass. The extracted oil from BSF larvae, rich in fatty acids (FA), offers an eco-friendly alternative for the cosmetic industry. In this study, larvae sourced from a Portuguese company were fed olive pomace, a by-product of olive oil production.

The lipidic sample extracted revealed a composition high in oleic acid, valuable for cosmetics. Investigating the biological activity of lipid extractions from larvae fed with olive pomace is a novel approach. Notably, the n-hexane ultrasound-assisted extraction method demonstrated potent antioxidant properties, and some extracts displayed antimicrobial activity. Five non-cytotoxic extracts; three with no relevant activity (IC_50_ from 236 to >400 μg/mL).

These findings highlight BSF larvae as an environmentally friendly source of fatty acids, offering promising alternatives for diverse applications.

## Highlights

• Billions of tons of food are globally wasted.
• BSF reduces waste, yields nutrient-rich resource for human uses.
• BSF larvae oil, rich in FA, promises eco-friendly cosmetic ingredient substitution.
• BSF larvae yield oleic acid, show diverse biological activities.
• Eco-friendly FA source with enhanced resilience.

## Graphical abstract

**Figure:**
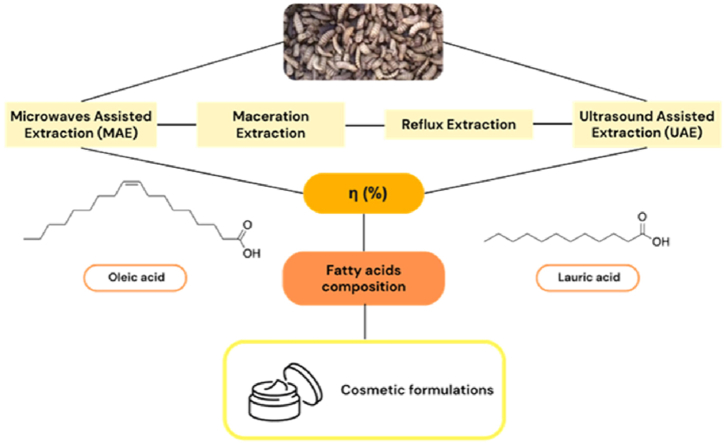

## 1 Introduction

Worldwide, an alarming 1.3 billion tonnes of food are wasted annually, posing significant concerns for the environment, economy, society, and public health [1]. As societies grapple with the multifaceted consequences of climate change, the urgency to address the issue of food waste becomes apparent, necessitating innovative solutions [2]. A substantial portion of the escalating global organic waste is attributed to food, posing threats to human health, biodiversity, and ecosystems [3,4]. In response to the surge in organic waste, the European Commission launched the "new circular economy action plan" in 2020 to promote a circular economy through the recycling and reuse of waste resources [5].

From this perspective, the cultivation of *Hermetia illucens* larvae presents a promising solution for effective waste biomass utilization. This sustainable approach allows for the conversion of organic waste into valuable larval biomass of high biological value, rich in essential nutrients [6,7]. The larval biomass comprises proteins (37 %–63 % of their dry weight), lipids (both saturated and unsaturated fatty acids, which can constitute over 40 % of the larval dry matter), as well as minerals and fibers [8]. Commonly known as the black soldier fly (BSF), *H. Illucens* exhibits unique feeding behaviour during its larval phase, consuming decaying organic materials and significantly reducing organic biomass. This process produces nutrient-dense resources that can be used primarily in animal feed, but also in the energy and cosmetic industries [9], and for extracting molecules of pharmacological interest [7,8,[10], [11], [12], [13]]. The efficiency of bioconversion is highly dependent on environmental conditions and the characteristics of the substrate [14,15]. Under optimal conditions, BSF larvae can reduce the substrate by up to 80 %, converting it into larval biomass. The timeframe for this conversion varies based on the specific rearing conditions [8].

Additionally, *H. Illucens* is rich in fatty acids (FA), and the most abundant are lauric, myristic, palmitic, and oleic acid [9,11]. These FA are typically produced through the hydrolysis and fractionation of vegetable and animal fats and oils. Lauric acid is primarily produced via saponification followed by fractional distillation; Palmitic acid is typically produced through distillation or crystallization after hydrolysis; Oleic acid production involves saponification and distillation; and Myristic acid is commercially produced through saponification and fractionation [9]. These FA, with diverse triglycerides and additional components (synthetic esters, fatty alcohols, silicones), have applications in the cosmetic sector for skincare purposes. FA soaps are commonly used as emollients for softening the skin. Indirectly, they hydrate the skin by reducing trans-epidermal water loss. The properties of fats can differ depending on the fatty acid profile. In Borrelli et al. [9,16] study, lauric acid and its monoglyceride derivative, monolaurin, demonstrate the strongest antimicrobial activity [9,16]. The fats contribute to cosmetic formulations for their emulsifying properties and enhancing viscosity. Simultaneously, unsaturated fatty acids play a crucial role in reinforcing the skin barrier, preventing moisture loss, supporting structural integrity affected by external factors and exhibiting anti-inflammatory properties [9]. The potential substitution of BSF-derived FAs for traditional sources, like coconut or palm kernel oil, presents an environmentally friendly alternative [9,11,17]. Given the similar FAs profile of BSFL is similar to that of palm kernel and coconut oil, its lipids are particularly well-suited for use in shower gels and soaps [17]. The environmental impact is substantial, as the rising demand for vegetable oils and biofuels leads to water waste, tropical deforestation, biodiversity loss, and habitat fragmentation. As a result, palm kernel and coconut oil face considerable criticism from an ecological perspective [16].

This paper aligns with the circular economy model bringing added value to both the food and cosmetic industries by offering a potential aid to two global issues. Portugal, a significant contributor to the EU's olive oil production, faces the challenge of olive pomace disposal [18,19].

Approximately 45 % of antioxidant compounds are indeed lost in olive pomace. Both olive and its by-products contain phenolic compounds that possess antimicrobial, anti-inflammatory, and chemo-preventive properties [20]. Recognizing the potential transfer of these properties to BSF larvae through larval feeding, underscores the importance of optimizing the larval diet for desired outcomes in the extracted compounds. Sourced from a Portuguese company committed to a circular economy, a pioneering effort to standardize and, in turn, reduce their own biological waste, lead to the implementation of olive pomace as the primary component for larvae feed. This strategic choice not only adds innovation to the experimental approach but also holds potential implications for optimizing larvae growth and development. Using olive pomace—a by-product of olive oil production—as a feed represents a novel exploration; it introduces a distinctive element to the study that contributes to the broader understanding of larval nutrition and its impact on biological activity results. BSF larvae, as a novel source of FA, prove to be not only more resilient but also more ecologically sound compared to current alternatives.

## 2 Material and methods

### 2.1 Sample origin and larvae preparation details

The batch of larvae were supplied by Ingredient Odyssey SA (EntoGreen) and were produced in an industrial unit located in Santarém, Portugal. Adult flies were kept in climate-controlled rooms with specific temperature, ventilation, light and humidity parameters, in order to promote adult reproduction. Fly eggs were dosed and incubated in a specific substrate for juvenile larvae for 5 days. Juvenile larvae were them inoculated in a vegetable by-product mixture and introduced in an automated warehouse with a climate control system. Larvae feed was produced in a feeding machine and formulated for an adequate nutrient level. The main constituent of the larvae feeding substrate was olive pomace which was mixed with other vegetable by-products.

Larvae feed nutrient value, ingredients and climate conditions are a trade secret and cannot be disclosed. Industrial parameters and outputs will not be disclosed.

The larvae underwent a thorough cleansing process, rinsing with running and distilled water twice. Afterward, the excess water was removed using paper. Subsequently, the larvae were placed in an oven set at 70 °C, for 48 h. Once dried, the larvae were stored in a desiccator for preservation.

### 2.2 Extraction methods

To optimize extraction efficiency and effectiveness, it was employed four techniques: reflux extraction, maceration, microwave-assisted extraction (MAE), and ultrasound-assisted extraction (UAE). The larvae were finely ground before extraction, conducted at a concentration of 10 % (w/v). Two organic solvents, acetone and *n*-hexane were used in separate batches, with distilled water exclusively for maceration and MAE.

#### 2.2.1 Reflux extraction

Reflux extraction, known for its efficiency [21], was performed using a reflux condenser. Five grams of larvae were mixed with 50 mL of either acetone or *n*-hexane in a round-bottom flask attached to the reflux system. The mixture was heated to 56 °C for acetone and 69 °C for *n*-hexane using a laboratory heating blanket, maintained for 3 min, and then cooled to room temperature.

#### 2.2.2 Maceration

Maceration, a straightforward but less efficient method, involved stirring the larvae with solvents for 2 h at room temperature. Despite its simplicity, maceration has the drawback of long extraction times and lower efficiency [21].

#### 2.2.3 Microwave-assisted extraction (MAE)

MAE was conducted using a microwave reaction system (Multiwave PRO, Anton Paar). Larvae samples mixed with *n*-hexane or acetone were placed into five glass vials with magnetic stirrers, loaded into the microwave chamber. The system's digital panel-controlled parameters like processing time and power, with maximum temperatures set at 50 °C for acetone and 60 °C for *n*-hexane. The procedure involved three 15-min cycles, replacing the solvent with fresh solvent between each cycle. MAE enhances extraction by aligning heat and mass transfer, accelerating yield, minimizing thermal degradation, and selectively heating plant materials, making it environmentally friendly [21].

#### 2.2.4 Ultrasound-assisted extraction (UAE)

UAE was performed using an ultrasound bath (Elmasonic Select 100, Elma Schmidbauer GmbH) under the same protocol of three 15-min cycles, with the solvent refreshed between each cycle. UAE uses cavitation to accelerate solute dissolution, diffusion, and heat transfer, enhancing extraction efficiency and reducing solvent and energy consumption [21].

#### 2.2.5 Solvent characteristics

*n*-hexane, a non-polar solvent, is effective in lipid extraction due to its lipid-solubilizing properties, whereas aqueous extractions depend on the insolubility of lipids in water. Acetone, a polar solvent, can dissolve both polar and nonpolar substances, making it versatile for lipid extraction. The choice of extraction method significantly affects both the yield, and the types of lipids extracted. Different techniques yield varying efficiencies and target specific lipid classes [22]. *n*-hexane is widely used commercially for extracting lipids from food and feedstuffs. However, it has several disadvantages: it is highly flammable, acutely toxic to aquatic life, and suspected of impairing fertility. Chronic exposure can damage organs, cause nerve disorders, and lead to skin and lung irritation [23].

#### 2.2.6 Post-extraction processing

After extraction, suspensions were filtered using vacuum filtration, and the solvents were evaporated at 40 °C using a rotary evaporator (Rotavapor R-100, BUCHI). The extraction yield was calculated following the equation:

$$Extraction\;yield\;\left(\%,w/w\right)=\frac{\text{lipidic}\;\text{extract}\;\text{weight}\;}{\text{dry}\;\text{weight}\;\text{of}\;\text{the}\;\text{larvae}}\;x\;100$$

### 2.3 Fatty acids composition

The analysis and quantification of fatty acid compositions within the larvae extracts were conducted following the previously described methodology [24]. In this procedure, 50 mg of each lipidic extracts were carefully weighed out. Subsequently, a solvent mixture comprising methanol, toluene, and sulfuric acid in a 2:1:1 ratio (v/v/v) was added in a quantity of 5 mL to the samples. These extracts were then subjected to overnight incubation within an agitated bath, maintaining a constant temperature of 50 °C. After the overnight incubation, 3 mL of distilled water was added to the mixture, followed by vertexing to ensure proper mixing. Then, 3 mL of ethyl ether was added and vortexed again. The resulting mixture was allowed to rest, allowing the phases to separate naturally. To remove any remaining water, sodium sulphate was utilized. The quantification of the lipid extract was conducted employing a YOUNG IN Chromass 6500 Gas chromatograph (GC) System instrument, which features a split/splitless injector, a flame ionization detector (FID), and a Zebron-Fame column. Identification and quantification of fatty acids were achieved by comparing the relative retention times of FAME peaks with those of commercial standards (standard mixture 47885-U, Sigma, St. Louis, USA). Data acquisition and processing were performed using Clarity DataApex 4.0 Software (Prague, Czech Republic), and the results were expressed in relative percentages of each fatty acid.

### 2.4 Evaluation of the extracts’ bioactivity

#### 2.4.1 Microorganisms used and growth conditions

Six selected strains, encompassing Gram-positive and Gram-negative bacteria as well as fungi were evaluated. The Gram-positive strains tested included *Staphylococcus aureus* (ATCC 25923), which was incubated aerobically for 24 h at 37 °C, and *Propionibacterium acnes* (ATCC 11827) along with *Staphylococcus epidermidis* (ATCC 13360), both of which were incubated anaerobically for 48 h at the same temperature. In contrast, the Gram-negative bacteria *Escherichia coli* (ATCC 25922) and *Pseudomonas aeruginosa* (ATCC 9027), and the fungal strain *Candida albicans* (clinical isolate), were incubated aerobically for 24 h at 37 °C.

#### 2.4.2 Antimicrobial assay

The antimicrobial activity of the lipidic extracts was evaluated using the microdilution method [25] and the rapid *p*-iodonitrotetrazolium chloride (INT) colorimetric assay to determine the minimum inhibitory concentration (MIC). For antibacterial activity, the lipid extracts were dissolved in mueller-hinton broth (MHB) with 0.5 % Tween 80, while for antifungal activity were dissolved in malt extract broth (MEB) and serially diluted in the same medium and tested up to a concentration of 2.5 % (v/v). Positive controls were tailored to the specific strains under study. Ketoconazole at 1 mg/mL was used for fungi; ampicillin at 10 mg/mL for anaerobic Gram-positive bacteria; vancomycin at 1 mg/mL for *Staphylococcus epidermidis*; and streptomycin at 1 mg/mL for Gram-negative bacteria and *Staphylococcus aureus*. Each assay was performed in duplicate to ensure reliability.

To determine the minimum bactericidal concentration (MBC), swabs were taken from wells after MIC testing and streaked onto agar plate. These plates were incubated for either 24 or 48 h at 37 °C. The MBC was identified was the lowest concentration where no visible microbial growth occurred, indicating bacterial death.

#### 2.4.3 Cell lines

RAW 264.7 (murine macrophage cell line) commercially acquired European Collection of Authenticated Cell Cultures (ECACC 91062702); ExPASy Cellosaurus database information: RRID: CVCL_0493. HFF-1 (primary human foreskin fibroblast) commercially acquired ATCC (SCRC-1041); ExPASy Cellosaurus database information: CVCL_3285. HaCaT (human keratinocytes) commercially acquired Leibniz - Institut DSMZ (ACC-771); ExPASy Cellosaurus database information: CVCL_0038.

Cell lines are purchased commercially from companies specialized in their commercialization and which guarantee their authenticity. All the precautions recommended by the suppliers are scrupulously followed. In addition to the precautions associated with laboratory practices in the manipulation of cell lines, such as aseptic conditions, prevention of cross-exchange, and change of culture medium, among others, the rate of cell multiplication is monitored on a routine basis so any changes in the rate of proliferation are noticeable. In addition, the cells are used for a maximum of 50 passages. Any modification in the cells’ multiplication rate or morphology are duly eliminated and replaced with new aliquots that are properly stored.

All cell lines were tested for the presence of mycoplasma, of which the results were negative.

#### 2.4.4 Antioxidant activity

The antioxidant activity of the lipid extracts was assessed through two cell-based assays. The lipid extracts were dissolved in either acetone or *n*-hexane, depending on the extraction solvent used, and then diluted in a range of 160 to 0.16 mg/mL (160, 80, 40, 20, 10, 5, 2.50, 1.25, 0.63, 0.31, and 0.16 mg/mL). The thiobarbituric acid reactive substance assay (TBARS) consists of the inhibition of lipid peroxidation in porcine (*Sus scrofa*) brain homogenates and was assessed by measuring the decrease in thiobarbituric acid reactive substances. The intensity of the color formed by malondialdehyde-thiobarbituric acid (MDA-TBA) was measured at 532 nm using absorbance. The results were reported as EC_50_ values (mg/mL), indicating the sample concentration that provides 50 % of the antioxidant activity [26].

Subsequently, the cellular antioxidant activity (CAA) was evaluated using a murine macrophage cell line (RAW 264.7). This assay measured the extracts' ability to prevent the oxidation of intracellular 7′-dichlorodihydrofluorescein (DCFH) following a previously described methodology. Quercetin was utilized as the positive control, while 7′-dichlorodihydrofluorescein and Dulbecco's modified Eagle's medium (DMEM) served as negative controls. The eight lipidic extracts were dissolved in DMSO:water (50:50, v/v), and tested at the final concentrations of 8, 2, 0.5 and 0.125 mg/mL. The results were expressed as a percentage inhibition of the formation of reactive oxygen species (ROS) at the maximum concentration tested [27].

#### 2.4.5 NO-production inhibition assay

The ability of the lipidic extracts to inhibit nitric oxide (NO) production in the murine macrophage cell line (RAW 264.7) was evaluated following the procedure previously described [28]. The eight lipid extracts were initially dissolved in a DMSO:water solution (50:50, v/v) and subsequently diluted to achieve a concentration range of 0.125–8 mg/mL for testing. 300 μL of the cell suspension of macrophages with a cell density of 5 × 10^5^ cells/mL was placed in each well. The microplate was incubated for 24 h in order to allow adequate adherence and multiplication of the cells. After, the cells were treated with different concentrations of oils (400, 100, 25 and 6.25 μg/mL) and incubated for 1 h. Stimulation was performed with the addition of 30 μL of the liposaccharide solution - LPS (1 mL/mL) and incubated for an additional 24 h. The vehicle solvent (DMSO:water, 50:50) was used as a negative control, with no LPS added to these wells. Dexamethasone (50 μM) served as a positive control. Quantification of nitric oxide was performed using a Griess reagent system kit and through the nitrite calibration curve (100 mM sodium nitrite at 1.6 mM) prepared in a 96-well plate. The nitric oxide produced was determined by reading absorbances at 540 nm (ELX800 Biotek microplate reader, Bio-Tek Instruments, Inc., Winooski, VT, USA) and by comparison with the standard calibration line. The results were calculated through the representation of the percentage of inhibition of nitric oxide production versus the sample concentration and expressed in relation to the concentration of each of the extracts that causes the 50 % inhibition of nitric oxide production - IC_50_. The outcomes were expressed as the extract concentration required for a 50 % inhibition of NO production (IC_50_, μg/mL) [29].

#### 2.4.6 Cytotoxic activity in skin cell lines

The cell viability determination after incubation with the eight lipidic extracts was adapted from the sulforhodamine B (SRB) protocol previously described [30]. Cell lines of fibroblasts (HFF-1) and keratinocytes (HaCaT) were used. The eight lipid extracts were initially dissolvent in DMSO and subsequently rediluted in distilled water to achieve the concentrations of 400, 200, 100 and 50 μg/mL. The data were analyzed using GraphPad Prism Software (GraphPad Prism version 10.0.2 for Windows, GraphPad Software, La Jolla, CA, USA), and all results were confirmed microscopically. The results were expressed as sample concentration required to inhibit a 50 % cell viability, IC_50_ mean ± SEM.

#### 2.4.7 Tyrosinase inhibition activity

The L-Dopa oxidase activity of mushroom tyrosinase (mTYR) was assayed for the lipidic extracts with some modifications following the method previously described [31]. As a positive control, 4-*n*-butylresorcinol, a potent tyrosinase inhibitor, was employed at concentrations ranging from 0 to 20 μM or 0–3.3 μg/mL. The assay was performed in a 96-well microplate as follows: (Samples) 7 μL of samples (100, 50, 25, 12.5 μg/mL dissolved in 1 % DMSO), 119 μL of phosphate buffer (PB; 0.1 M, pH 6.8), 7 μL of tyrosinase enzyme (mTYR, 142 units/mL), and 67 μL of L-DOPA (5 mM); (Control, 100 % enzymatic activity) 7 μL of solvent samples, 119 μL of PB, 7 μL of mTYR, and 67 μL of L-DOPA; and (background control) 133 μL of PB and 67 μL of L-DOPA. In kinetic mode, the mixtures were measured (475 nm) at 37 °C using a microplate reader (FLX800 Biotek).

The data were analyzed using GraphPad Prism Software, calculating the percentage inhibition of the tested samples by normalizing the ABS data using both controls and determining it from the slope of the kinetic curve that presented a better fit for the data.

### 2.5 Statistical analyses

All experiments were performed in triplicate and duplicate (microbiology activity). The results were presented as the mean value ± standard deviation (except for antimicrobial activity). The obtained results were analyzed using GraphPad Prism (Prism for Mac OS, Version 10.1.1 (270); GraphPad Software, 225 Franklin Street. Fl. 26 Boston, MA 02110) by applying one-way analysis of variance (ANOVA) followed by Tukey's HSD test, with α = 0.05.

## 3 Results and discussion

### 3.1 Extraction yield

**Fig. 1:**
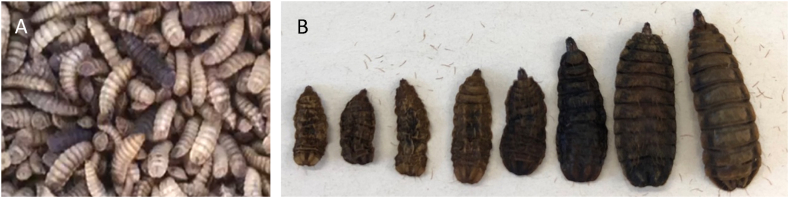
A) Live larvae in the excrement; B) Dead larvae after washing and drying in the oven.

The initial phase of the extraction involved cleansing the larvae since they were delivered in excrement. The larvae were washed and subsequently dried to eliminate excess water from the washing process (Fig. 1).

**Graph 1:**
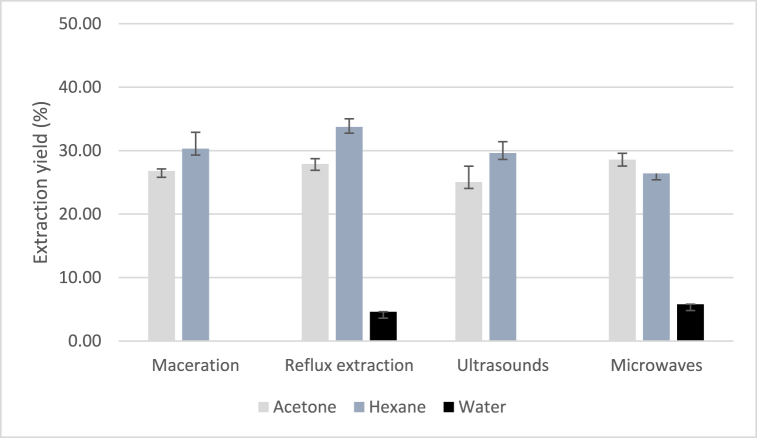
Extraction yield (%) of the lipidic extracts of dried BSF larvae. Yield calculated as the ratio of dry weight of the larvae to lipidic extract weight (w/w). Extractions performed at 10 % (w/v) with different solvents and methods.

The extraction of BSF larvae was conducted using four different methods: maceration, reflux extraction, UAE and MAE. Three solvents—distilled water, acetone, and *n*-hexane—were employed. The extraction yields (%) were calculated as the ratio of the dry weight of the larvae to the weight of the lipidic sample weight (w/w). Graph 1 illustrates the yields obtained, ranging from 4.60 % to 33.75 % (w/w). The *n*-hexane reflux extraction method achieved the highest extraction yield of 34 % (w/w) while the acetone MAE method showed the highest extraction yield, reaching 29 % (w/w).

As it is well-established, organic solvents, based on their polarity, have the capacity to extract diverse classes of compounds. This phenomenon serves as a rationale for the superior performance observed with *n*-hexane and acetone in comparison to alternative extraction techniques involving water [32].

Due to the limited extraction yields observed in aqueous extracts, we proceeded to evaluate the biological activities with organic solvents extracts.

### 3.2 Fatty acids composition

The fatty acid composition of the lipidic extracts from BSF larvae was examined, with the results were expressed in relative percentages as shown in Table 1. Oleic acid (C18:1n9c) was the most prevalent fatty acid, comprising 30–36 % of the total composition. Lauric acid (C12:0) followed at 21–37 %, and palmitic acid (C16:0) at 15–19 %. The exception was the acetone MAE extract, where lauric acid was predominant at 37 %, and oleic acid was secondary at 24 %.

**Table 1:** Relative percentage of fatty acids composition of the lipidic extracts of BSF larvae (mean ± SD, *n=3*).

	*n*-Hexane extractions				Acetone extractions			
	Maceration	Reflux extraction	UAE	MAE	Maceration	Reflux extraction	UAE	MAE
Fatty acids	Relative percentage (%)							
**C6:0** Caproic acid	0.22 ± 0.02	0.15 ± 0.01	nd	0.184 ± 0.001	0.14 ± 0.01	0.23 ± 0.01	0.20 ± 0.01	0.62 ± 0.02
**C8:0** Caprylic acid	nd	nd	nd	0.15 ± 0.01	nd	nd	nd	0.23 ± 0.01
**C10:0** Capric acid	1.00 ± 0.09	0.94 ± 0.07	0.88 ± 0.02	1.08 ± 0.03	0.84 ± 0.02	1.02 ± 0.03	0.89 ± 0.02	1.49 ± 0.01
**C12:0** Lauric acid	26 ± 1	26 ± 1	25.7 ± 0.6	30.1 ± 0.4	21.29 ± 0.03	26.1 ± 0.8	22.8 ± 0.3	37.0 ± 0.8
**C14:0** Myristic acid	5.3 ± 0.2	5.7 ± 0.1	5.92 ± 0.06	5.73 ± 0.02	4.55 ± 0.05	5.4 ± 0.2	4.85 ± 0.06	7.1 ± 0.1
**C14:1** Myristoleic Acid	0.19 ± 0.01	0.20 ± 0.01	nd	0.23 ± 0.01	0.173 ± 0.004	0.18 ± 0.01	0.158 ± 0.001	0.18 ± 0.02
**C15:0** Pentadecylic acid	0.25 ± 0.01	0.21 ± 0.01	nd	0.14 ± 0.01	0.174 ± 0.002	0.20 ± 0.01	0.175 ± 0.002	0.31 ± 0.02
**C15:1** Pentadecenoic acid	0.125 ± 0.001	0.120 ± 0.004	nd	0.08 ± 0.01	0.11 ± 0.01	0.118 ± 0.002	0.101 ± 0.003	0.14 ± 0.01
**C16:0** Palmitic acid	17.70 ± 0.06	17.59 ± 0.05	18.4 ± 0.2	16.0 ± 0.2	15.0 ± 0.3	15.7 ± 0.2	16.3 ± 0.1	18.7 ± 0.3
**C16:1** Palmitoleic acid	2.17 ± 0.03	2.03 ± 0.02	1.79 ± 0.08	1.70 ± 0.02	2.10 ± 0.04	2.05 ± 0.03	1.94 ± 0.04	1.59 ± 0.04
**C17:0** Margaric acid	0.36 ± 0.01	0.32 ± 0.01	nd	0.207 ± 0.004	0.32 ± 0.01	0.324 ± 0.001	0.311 ± 0.003	0.35 ± 0.02
**C18:0** Stearic acid	3.29 ± 0.02	3.3 ± 0.1	3.38 ± 0.02	2.67 ± 0.05	3.23 ± 0.06	3.03 ± 0.09	3.16 ± 0.02	3.00 ± 0.02
**C18:1n9c** Oleic acid	35 ± 1	30.4 ± 0.9	31.0 ± 0.8	30.2 ± 0.5	36.21 ± 0.05	31.6 ± 0.8	34.29 ± 0.05	23.9 ± 0.2
**C18:2n6c** Linolenic acid	8.05 ± 0.01	11.8 ± 0.4	11.74 ± 0.04	10.6 ± 0.3	14.3 ± 0.3	12.8 ± 0.3	13.34 ± 0.08	5.02 ± 0.01
**C18:3n3** α-linolenic acid	0.53 ± 0.01	1.25 ± 0.06	1.3 ± 0.1	0.88 ± 0.02	1.56 ± 0.03	1.38 ± 0.04	1.48 ± 0.01	0.34 ± 0.03
SFA	54±1^a,c^	54±1^a,c^	54.2 ± 0.8^a,c^	56.3 ± 0.75^a^	45.6 ± 0.2^b^	52±1^c^	48.7 ± 0.2^d^	68.9 ± 0.3^e^
MUFA	38±1^a^	32.8 ± 0.9^b^	32.8 ± 0.7^b^	32.2 ± 0.5^b^	38.60 ± 0.08^a^	33.9 ± 0.8^b^	36.5 ± 0.1^a^	25.8 ± 0.3^c^
PUFA	8.58 ± 0.01^d^	13.0 ± 0.5^a^	13.0 ± 0.2^a^	11.0 ± 0.2^e^	15.8 ± 0.3^b^	14.2 ± 0.4^c^	14.82 ± 0.07^b,c^	5.36 ± 0.02^f^

UAE-ultrasound assisted extraction; MAE-microwaves assisted extraction; SFA-saturated fatty acids; MUFA-monounsaturated fatty acids; PUFA-polyunsaturated fatty acids; nd-not detected. Statistical differences between extraction methods were assessed using ANOVA followed by Tukey's post-hoc test. In row, different letters (a-f) next to values indicate significant differences at *p* < 0.05.

Several authors have described that the type of substrate that BSF is fed influences both the development and biochemical composition of the larvae [12,33]. In the study conducted by Ameixa et al. [18], varying percentages of olive pomace were tested as feed for BSF larvae. The findings indicated that an increased quantity of olive pomace in the larvae's diet correlated with higher oleic acid composition (22–57 %). Similarly, Starčević et al. [34] investigated the impact of feeding media on the growth and fatty acid composition of BSF larvae. They compared larvae fed with crude olive cake (COC) and processed animal protein (PAP). Larvae fed with olive pomace exhibited a reduction in lauric acid and saturated fatty acids, alongside an increase in oleic acid and monounsaturated fatty acids (MUFA). These findings highlight the significant influence of diet on the nutritional profile of BSF larvae, particularly in terms of fatty acid composition.

Since the batch of BSF larvae used in this study was fed the olive pomace, the results obtained were expected and confirm previous research results [18]. Oleic acid, known for activating lipid metabolism and enhancing skin barrier restoration, featured prominently, making it suitable for cosmetic product formulations [35]. Lauric acid, renowned for its antimicrobial and antibacterial properties, is a significant component in the production of soaps, creams, and various cosmetics [35]. Palmitic acid (C16:0), the third most prevalent fatty acid, serves as a structural component, emulsifier, and emollient in cosmetic products [35]. Additionally, myristic acid (C14:0) is commonly used in cosmetic formulations for thickening and stabilizing emulsions. It also helps restore the cutaneous barrier properties and enhances the permeability of active components into the skin [33]. In conclusion, these results suggests that BSF larvae from circular economies could serve as a valuable source of fatty acids for cosmetic product formulations.

Based on the existing literature, which highlighted the impact of larval diet on fatty acid composition, our study seamlessly progresses to investigate the biological activities of BSF lipid extracts abundant in oleic acid. The obtained results not only align with previously published findings regarding the influence of larval feed on fatty acid composition but also extend the exploration into the functional aspects of these lipid samples.

### 3.3 Antimicrobial activity

The antimicrobial activity of BSF larvae lipidic extracts is crucial for their potential applications across diverse fields such as pharmaceuticals, cosmetics, and food preservation. Gram-positive and Gram-negative bacteria, along with yeasts, are common pathogens that pose significant health risks. Developing effective antimicrobial agents derived from natural sources like BSF larvae could provide sustainable alternatives to synthetic antibiotics and antimicrobials.

**Graph 2:**
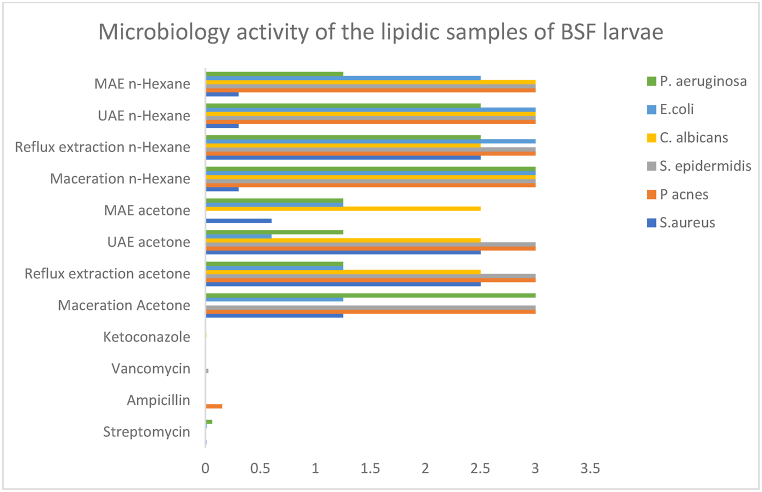
Microbiology activity of the lipidic extracts of BSF larvae. Positive controls: Vancomycin, Streptomycin, Ketoconazole tested at 1 mg/mL, and Ampicillin tested at 10 mg/mL. UAE-ultrasound assisted extraction; MAE-microwaves assisted extraction.

In this study, the MIC and MBC of each extract were determined. MIC indicates the lowest concentration of an extract needed to inhibit visible growth of microorganisms, while MBC denotes the lowest concentration required to kill the microorganisms. By testing against both Gram-positive and Gram-negative bacteria, as well as yeasts, we aimed to assess the broad-spectrum antimicrobial potential of these extracts. Graph 2 presents the results of the antimicrobial activity assay.

The extracts obtained through *n*-hexane maceration, *n*-hexane UAE, *n*-hexane MAE and acetone MAE demonstrated moderate activity against Gram-positive bacteria, namely *S. aureus*. The acetone maceration extract exhibited moderate activity against fungi, specifically *C. albicans,* while acetone UAE demonstrated moderate activity against Gram-negative bacteria, particularly *E. coli*. These results align with current literature, which indicates that Gram-positive bacteria, characterized by a single thick peptidoglycan layer, are more sensitive to fatty acids (FAs) than Gram-negative species. This structural difference allows intermediate- and long-chain FAs to penetrate Gram-positive bacteria more effectively, thereby exerting their toxic effects [36].

Previous studies have demonstrated that *H. illucens* larvae reared on decomposing organic environments—rich in diverse microorganisms such as bacteria and fungi [37,38]—exhibit antimicrobial activity against several Gram-positive and Gram-negative microorganisms [33,37]. This highlights their innate defense mechanisms against microbial threats, potentially involving antimicrobial peptides (AMPs) and other bioactive substances [[37], [38], [39]], as supported by the findings of Vogel et al. [38], who observed heightened AMP expression in larvae fed high-bacterial-load diets.

Studies have highlighted that saturated FA like capric acid (C10:0) and lauric acid (C12:0) exhibit broad antimicrobial activities [40]. Additionally, butyric acid (C4:0), capric acid (C10:0), palmitoleic acid (C16:0), α-linolenic acid (C18:3 n3), and eicosenoic acid (C20:1) have also demonstrated significant antimicrobial properties [36]. Furthermore, a significant finding connection between the antimicrobial properties and the presence of lauric acid [16,41].

Franco et al. [36] demonstrated that administering various by-products from agricultural and zootechnical chains can influence the antimicrobial activity of BSF larvae lipids.

In our study, BSF larvae were fed with olive pomace enriched in oleic acid, resulting in lipid extracts with higher concentrations of oleic acid. This finding aligns with the observed antimicrobial properties of the extracts, indicating the potential influence of dietary sources on the bioactivity of BSF larvae lipids.

### 3.4 Antioxidant activity

Skin cells are continuously exposed to harmful free radicals originating from both internal metabolic processes and external environmental factors. These reactive oxygen species (ROS) pose a significant threat as they can overwhelm the skin's natural defense mechanisms. Excessive ROS can damage the skin by targeting essential components such as sebum lipids, ceramides in the stratum corneum, and polyunsaturated fatty acids in cell membranes. This oxidative stress leads to cellular dysfunction and exacerbates skin aging processes [42].

To evaluate the protective potential of the lipidic extracts derived from BSF larvae, two distinct methods to assess antioxidant activity were employed: thiobarbituric acid reactive substances (TBARS) and the cellular antioxidant activity (CAA). These methods are well-established for measuring the ability of compounds to neutralize free radicals and protect against oxidative damage at both the molecular and cellular levels. The findings regarding the antioxidant capacity of the investigated samples are shown in Table 2.

**Table 2:** Antioxidant activity of the lipidic extracts of BSF larvae (mean ± SD, *n* = 3).

Samples		TBARS (EC_50_, mg/mL)	CAA (% inhibition of oxidation at 2 mg/mL)
**Acetone extractions**	**Maceration**	>106.7	69 ± 6^a^
	**Reflux extraction**	>106.7	62 ± 5^a^
	**UAE**	>133.3	62 ± 3^a^
	**MAE**	>106.7	77 ± 5^a^
***n*-Hexane extractions**	**Maceration**	>166.7	69 ± 9^a^
	**Reflux extraction**	11.6 ± 1.2^a^	64 ± 5^a^
	**UAE**	5.6 ± 0.1^b^	75 ± 3^a^
	**MAE**	>106.7	64 ± 3^a^
**Positive control**		0.0058 ± 0.0006	95 ± 5

Positive controls: Trolex (TBARS); Quercetin (CAA) at 0.3 μg/mL. UAE-ultrasound assisted extraction; MAE-microwaves assisted extraction. Statistical differences between extraction methods were assessed using ANOVA followed by Tukey's post-hoc test. In column, different letters (a, b) next to values indicate significant differences at *p* < 0.05.

The TBARS assay is used to detect lipid oxidation by measuring malondialdehyde (MDA), a by-product formed from the oxidation of unsaturated fatty acids. Samples were tested at different concentrations, and the results were expressed as EC_50_ values, indicating the concentrations in mg/mL at which the samples exhibited 50 % of their antioxidant activity. The samples *n*-hexane UAE and reflux extraction demonstrates moderate antioxidant activity in this method, whereas the remaining extracts did not exhibit significant activity.

On the other hand, the CAA technique assesses the ability of compounds to prevent the formation of DCF by 2,2′-azobis(2-amidinopropane) dihydrochloride (ABAP)-generated peroxyl radicals in RAW 264.7 macrophage cell line. Samples were tested across a range of concentrations to determine the inhibition concentration, measuring the percentage of cells protected from oxidation. Additionally, an IC_50_ value was calculated, representing the minimum concentration necessary to prevent 50 % of oxidation. The samples, acetone MAE and *n*-hexane UAE displayed the highest antioxidant activity, registering activity levels of 77 % and 75 %, respectively.

Previous studies have noted that protein hydrolysates derived from *H. illucens* larvae exhibit antioxidant activity. Riolo et al. [43] further highlighted those processes used in producing these hydrolysates, including the methods used to kill the larval, as well as chemical actions, can significantly impact the antioxidant properties of BSF hydrolysates. In contrast, the oil extracts in our study were not hydrolyzed, which may explain their moderate or lack of antioxidant activity. This suggests that hydrolysis may be a critical factor in enhancing the antioxidant potential of BSF-derived products.

### 3.5 NO-production inhibition assay

The ability of the extracts to inhibit the production of the pro-inflammatory mediator NO in LPS-stimulated murine macrophage cell line (RAW 264.7) was measured. The results obtained from this analysis are presented in Table 3.

**Table 3:** NO-production inhibition assay of the lipidic extracts of BSF larvae (mean ± SD, *n* = 3).

Samples		NO Production Inhibition (IC_50_, μg/mL)
**Acetone extractions**	**Maceration**	76 ± 2^c,d^
	**Reflux extraction**	113 ± 9^a^
	**UAE**	41 ± 1^b^
	**MAE**	48 ± 4^b^
***n*-Hexane extractions**	**Maceration**	145 ± 6^f^
	**Reflux extraction**	118 ± 3^a^
	**UAE**	80 ± 3^c,e^
	**MAE**	75 ± 3^d,e^
**Dexamethasone**		6.30 ± 0.4

IC_50_ results expressed in μg/mL. Results are expressed as mean ± standard deviation. Positive control: Dexamethasone. UAE-ultrasound assisted extraction; MAE-microwaves assisted extraction. Statistical differences between extraction methods were assessed using ANOVA followed by Tukey's post-hoc test. In column, different letters (a, f) next to values indicate significant differences at *p* < 0.05.

All the lipidic extracts of BSF larvae demonstrated anti-inflammatory activity. The most efficient sample was acetone UAE with IC_50_ = 41 ± 1 μg/mL, followed by acetone MAE that showed a IC_50_ = 48 ± 4 μg/mL. Although there are limited studies on BSF larvae tested for anti-inflammatory activity using the RAW 264.7 cell line, these results align with findings by Xiaoyan et al. [44], which reported anti-inflammatory activity through the reduction of NO levels in RAW 264.7 cells.

Fatty acids are known to modulate inflammation [45,46]. In our study, the lipidic extracts prominently featured oleic acid as the major FA. Pegorano et al. [47] demonstrated that semisolid dosage forms containing oleic acid exhibit anti-inflammatory effects *in vivo* via glucocorticoid receptors in a UVB-radiation-induced skin inflammation model. The anti-inflammatory effect of oleic acid is comparable to that of dexamethasone but without the adverse effects commonly associated with glucocorticoids. Oleic acid, as a natural compound, shows promise as a potential treatment option for inflammatory skin disorders, offering therapeutic benefits without the undesired side effects associated with traditional glucocorticoids [47,48].

### 3.6 Cytotoxic effects on skin cell lines

The evaluation of cytotoxicity using HaCaT and HFF-1 refers to testing the potentially harmful effects of the lipidic extracts on two different cell lines. The cytotoxicity studies involve exposing these cell lines to various concentrations of larvae lipidic extracts to determine how they affect the cells' viability and health. The *n*-hexane MAE extract showed moderate cytotoxicity against HFF-1 with an IC_50_ of 236 μg/mL. The remaining samples did not exhibit cytotoxicity higher than 50 % at the concentrations tested or have an IC_50_ close to the maximum concentration tested (Table 4).

**Table 4:** Cytotoxicity results against different cell lines. IC_50_ results are expressed in μg/mL.

Cytotoxicity (IC_50_ μg/mL)			
Samples		HaCaT	HFF-1
**Acetone extractions**	**Maceration**	>400	>400
	**Reflux extraction**	399 ± 12^a^	>400
	**UAE**	>400	343 ± 25^a^
	**MAE**	>400	>400
***n*-Hexane extractions**	**Maceration**	>400	>400
	**Reflux extraction**	>400	>400
	**UAE**	>400	>400
	**MAE**	>400	236 ± 15^b^

Cytotoxicity of HaCaT (Human keratinocytes) and HFF-1 (Fibroblasts) cells. Samples tested between 50 and 400 μg/mL. IC_50_ ± SEM. Statistical differences between extraction methods were assessed using ANOVA followed by Tukey's post-hoc test. In column, different letters (a, b) next to values indicate significant differences at *p* < 0.05.

In a previous study, Muangrat et al. [49] demonstrated that supercritical CO_2_-extracted oil samples exhibited significant inhibition of HaCaT growth from 0.01 to 100 mg/mL. It is essential to highlight that the concentrations investigated in their research were considerably higher than those examined in our study. While these findings hold promise for further research, the elevated concentrations may prompt safety concerns. There is no assurance that such high concentrations will not lead to adverse effects when applied to the skin, warranting careful consideration in future investigations.

Phongpradist et al. [46] conducted MTT cell viability assays on HaCaT keratinocytes, primary human dermal fibroblasts, and peripheral blood mononuclear cells, reporting no cytotoxic effects of BSF larvae oil with IC_50_ values exceeding 200 μg/mL.

### *3.7* *In vitro* skin enzyme

#### 3.7.1 Tyrosinase inhibition activity

Currently, many cosmetic products integrate not only antioxidant but also anti-pigmenting agents such as kojic acid, linoleic acid, hinokitiol, renowned for their skin-whitening effects [46,50]. Tyrosinase is an important enzyme that catalyzes the formation of 3,4-dihydroxyphenylalanine (L-DOPA) by hydroxylation of L-tyrosine and oxidases L-DOPA to dopaquinone; that contributes to human skin's pigmentation process [51]. FA are integral constituents of the epidermal layer, playing a crucial role in regulating melanogenesis through biosynthetic pathways. Previous studies described that the saturation of the FA is essential to determine the pigmentation. Saturated fatty acids, such as palmitic acid (C16:0) and stearic acid (C18:0), enhance pigmentation. On the other hand, unsaturated fatty acids including oleic acid (C18:1), linoleic acid (C18:2), and α-linolenic acid (C18:3) supresses melanogenesis and tyrosinase activity [51,52].

Phongpradist et al. [46] studied the fatty acid profile of BSF larvae oil, identifying linoleic acid as the predominant fatty acid, followed by oleic and palmitic acids. They indicated that BSF larvae oil significantly reduced melanin content (78 % inhibition). Linoleic acid is known to inhibit melanogenesis by reducing the levels of tyrosinase, primarily through enhanced ubiquitination and subsequent degradation by the proteasome. Additionally, it promotes epidermal turnover and enhances the shedding of melanin from the epidermis [[53], [54], [55], [56]]. Hwang et al. [57] explored the fatty acid composition of BSF larvae oil, finding lauric acid to be the most abundant fatty acid, with oleic and palmitic acids also present. Their research further evaluated the skin-whitening potential of *H. illucens* oil (HIO) and its fermented variant (FHIO). Results showed that FHIO exhibited a significantly higher tyrosinase inhibition rate of 28.69 %, compared to 24.68 % for HIO, indicating that fermentation boosts the oil's tyrosinase inhibitory effect. Impressively, the inhibition rate of FHIO was nearly equivalent to that of kojic acid at 32.58 %, highlighting the promising skin-whitening capabilities of fermented BSF oil.

**Graph 3:**
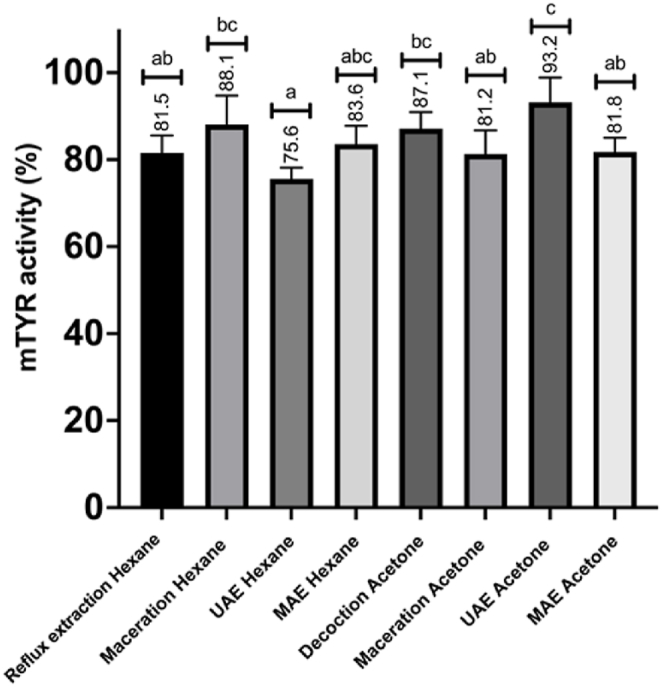
Percentage of mushroom tyrosinase enzyme (mTYR) activity of the different samples (sample concentration 100 μg/ml). Statistical differences were assessed by one-way ANOVA and Tukey's HSD post hoc test (α = 0.05): lower-case letters indicate significant differences among samples.

In our experiments, the lipid extracts inhibited the mushroom tyrosinase enzyme at the tested concentrations. As shown in Graph 3, the *n*-hexane UAE sample achieved the highest inhibition, reducing tyrosinase activity to approximately 75.6 % (around 25 % inhibition), whereas acetone UAE exhibited the lowest, with less than 10 % inhibition.

From a different perspective, the consumption of fatty acids by humans has been explored for its potential skin benefits. Cosgrove et al. [58] findings indicated that increased dietary intake of linoleic acid could play a protective role against senile dryness and skin atrophy in middle-aged women. Linoleic acid, classified as an omega-6 polyunsaturated fatty acid (PUFA), is considered essential as it cannot be synthesized by the body and must be obtained through diet. Once ingested, linoleic acid undergoes conversion to other beneficial PUFAs such as eicosapentaenoic acid (EPA) and docosahexaenoic acid (DHA).

## 4 Conclusion

*Hermetia illucens,* commonly known as Black Soldier Fly (BSF), has emerged as a pivotal species in sustainable waste management and resource utilization. This study has unveiled significant findings that highlight the potential applications of BSF lipidic extracts in diverse fields, particularly cosmetics and biomedical sciences. Sourced from a Portuguese company committed to sustainable food practices, BSF larvae were subjected to innovative dietary standardization using olive pomace, a by-product of olive oil production.

The study employed maceration, reflux extraction, ultrasound-assisted extraction (UAE), and microwave-assisted extraction (MAE) methods, revealing varying efficiencies in obtaining lipidic-rich extracts from BSF larvae. Notably, n-hexane reflux extraction and acetone MAE yielded the highest extraction, highlighting their efficacy in obtaining lipid-rich extracts.

The fatty acid composition analysis revealed oleic acid (C18:1n9c) as the predominant fatty acid across most extracts, with variations observed based on the extraction method. This fatty acid profile aligns with previous studies correlating larval diet, particularly rich in oleic acid sources like olive pomace, to the composition of BSF lipids. These findings underscore the influence of larval diet on the biochemical composition of BSF lipids and their potential downstream applications.

Biological activity assays further demonstrated the diverse functional properties of BSF lipid extracts. They exhibited antioxidant activity, as evidenced by their ability to scavenge free radicals, and moderate antimicrobial properties against both Gram-positive and Gram-negative bacteria. Moreover, the extracts showed promising anti-inflammatory effects by reducing NO production in RAW 264.7 cells, indicative of their potential therapeutic utility in treating inflammatory skin disorders.

Furthermore, the evaluation of cytotoxicity on keratinocytes (HaCaT) and human dermal fibroblasts (HFF-1) revealed minimal adverse effects, with most extracts demonstrating no cytotoxicity profiles, ensuring their safety for potential cosmetic and biomedical applications.

Importantly, the inhibition of tyrosinase enzyme activity by BSF lipid extracts suggests their role in skin-whitening applications. This effect, particularly notable in extracts rich in oleic acid, highlights their potential as alternatives to conventional skin-whitening agents.

In conclusion, this study provides compelling evidence supporting the multifaceted bioactivity and cosmetic potential of BSF larvae lipid extracts. These findings pave the way for future research focusing on optimizing extraction methods, elucidating underlying mechanisms of bioactivity, and exploring novel applications in skincare and therapeutic formulations. The sustainable sourcing of BSF larvae and their lipidic extracts underscores their role in promoting circular economy principles and sustainable development in the cosmetics and biomedical industries.

## Funding sources

This work was supported by Fundação para a Ciência e Tecnologia (10.13039/501100001871FCT, Portugal) under the projects DOI 10.54499/UIDP/04567/2020 and DOI 10.54499/UIDB/04567/2020 (https://doi.org/10.54499/UIDP/04567/2020) attributed to CBIOS; for financial support through national funds 10.13039/501100001871FCT/10.13039/501100006111MCTES (PIDDAC) to 10.13039/501100004591CIMO (UIDB/00690/2020 and UIDP/00690/2020) and SusTEC (LA/P/0007/2020), and to the projects UIDB/04539/2020 and UIDP/04539/2020; national funding by 10.13039/501100001871FCT, P.I., through the institutional scientific employment program-contract for R. Calhelha contracts. We extend our gratitude to Professor Adília Januário Charmier (Lusófona University) for generously providing us with the microwave equipment essential for conducting the extractions.

## CRediT authorship contribution statement

**Márcia Santos Filipe:** Writing – original draft, Methodology, Investigation. **Rossana V.C. Cardoso:** Investigation. **Manuel Ayuso:** Investigation. **Daniel Murta:** Visualization. **Ana María Díaz-Lanza:** Visualization. **Catarina Rosado:** Visualization. **Tânia C.S.P Pires:** Investigation. **Ricardo C. Calhelha:** Writing – original draft, Supervision, Methodology. **Patricia Rijo:** Writing – review & editing, Validation, Supervision, Methodology, Funding acquisition, Conceptualization.

## Declaration of competing interest

The authors declare that they have no known competing financial interests or personal relationships that could have appeared to influence the work reported in this paper.
